# Association of Gene Polymorphism of Matrix Metalloproteinase-9 With Chronic Periodontitis: A Cross-Sectional Survey in a South Indian Population

**DOI:** 10.7759/cureus.91667

**Published:** 2025-09-05

**Authors:** Shahana C Mohamed, Raseena Beevi N, Moinak Banerjee, Sanish Sathyan, Bindu R Nayar, Presanthila Janam

**Affiliations:** 1 Department of Periodontics, Government Dental College, Thiruvananthapuram, Kerala University of Health Sciences, Thrissur, IND; 2 Human Molecular Genetics Laboratory, Rajiv Gandhi Centre for Biotechnology, Thiruvananthapuram, IND

**Keywords:** chronic periodontitis, gene polymorphism, indian, matrix metalloproteinase-9, periodontal disease, single-nucleotide polymorphism

## Abstract

Aims: Matrix metalloproteinases (MMPs) have an important role in the remodeling and destruction of periodontal tissues. Genetic polymorphisms of MMPs have been investigated as probable candidate gene markers, potentially associated with the susceptibility, severity, and clinical outcome of periodontal disease.

This cross-sectional study was designed to determine whether there is any association between the single-nucleotide polymorphism (SNP) of the *MMP9 *rs3918242 gene (promoter) with chronic generalized periodontitis as measured by polymerase chain reaction (PCR) direct sequencing, and to determine whether there is any association between the SNP of the *MMP9 *rs17576 gene with chronic generalized periodontitis as measured by PCR-restriction fragment length polymorphism (RFLP).

Methods: A total of 170 subjects participated in the study, which included 80 subjects with chronic generalized periodontitis and 90 controls, who belonged to the South Indian (Kerala) ethnicity. The samples were genotyped by using PCR followed by sequencing/RFLP. The genotype and allele frequency of the *MMP9 *gene were determined in both the cases and controls. A p value of <0.05 was considered statistically significant.

Results: In our study population, there was no statistically significant difference in the genotype frequencies of the *MMP9 *rs3918242 gene (p = 0.651) and no statistically significant difference in the allele frequencies (p = 0.808; odds ratio = 0.938, 95% CI: 0.562-1.567) between cases and controls. However, there was a statistically significant difference between genotype (p = 0.033) frequencies of *MMP9 *rs17576 in cases and controls.

Conclusion: Within the limitations of sample size and selection, results of the present study suggest a possible lack of association between the *MMP9* promoter gene polymorphism and susceptibility to chronic generalized periodontitis in the population studied. However, there might be an association between the genotype of the *MMP9* rs17576 gene polymorphism and susceptibility to chronic generalized periodontitis. Therefore, further research in this locus with larger samples with age- and gender-matched controls could provide important insights into the pathogenesis of periodontitis, identifying individuals at high risk.

## Introduction

Periodontal disease, a chronic immunoinflammatory disease caused by complex interactions between periodontal pathogens, their byproducts, and host responses, is one of the most important oral health problems worldwide. It stimulates the host's defense mechanisms to produce a variety of biologically active molecules designed to neutralize the bacterial attack. Although both environmental and microbiological variables cause and control periodontal disease, each person's genetic background affects how they react to comparable environmental stressors. Numerous studies have examined the link between gene polymorphisms and periodontitis susceptibility, which has prompted researchers to look for genetic markers of the disease.

Matrix metalloproteinases (MMPs) are a class of metal-dependent enzymes that play a crucial role in degrading proteins within the extracellular matrix and basement membranes during embryonic development, the morphogenesis stage, and the remodeling of tissues [1]. Various host cells, including macrophages, polymorphonuclear leukocytes, fibroblasts, bone cells, epithelial cells, and endothelial cells, secrete or release MMPs [2]. The existence of endogenous tissue inhibitors of metalloproteinases and the activation of latent proforms are prerequisites for MMP activity [1]. Inflamed periodontal tissues include a variety of MMP types. A lack of balance among MMPs and their host inhibitors can result in periodontal disease by initiating the degradation of structural proteins [3,4]. Several studies have supported the link between MMPs and periodontitis by demonstrating the presence of elevated levels of MMP-1, -2, -3, -7, -8, and -9 in tissues and gingival crevicular fluid of chronic periodontitis patients compared to healthy controls [5-9].

MMP-9, also known as 92-kDa type IV collagenase or gelatinase B, is required for the degradation of periodontal tissues in periodontitis [1,10-13]. In addition to denatured collagens (gelatin), MMP-9 is one of the MMPs that has activity against type IV, V, and XI collagens as well as proteoglycans and elastin [10,14]. A single-nucleotide polymorphism (SNP) is a small genetic change, or variation, that can occur within a person's deoxyribonucleic acid (DNA) sequence. The *MMP9 *gene'sSNP at position-1562 results from a C-to-T substitution [7]. Studies conducted in vitro have demonstrated that C-to-T substitution increases transcriptional activity in macrophages and causes a loss of nuclear protein binding to this region of the *MMP9* gene promoter. These in vitro findings are supported by the fact that people with the T allele had higher plasma levels of MMP-9 [7].

Given the critical role of MMP-9 in periodontal diseases, it is hypothesized that genetic variations influencing its expression or activity may affect an individual's susceptibility to and severity of the condition [10,13]. Hence, this study was carried out to investigate the association, if any, between the *MMP9* gene polymorphism and chronic generalized periodontitis. The objective of this cross-sectional study was to determine whether there is any association between the SNP of the *MMP9* rs3918242 gene (promoter) with chronic generalized periodontitis as measured by polymerase chain reaction (PCR) direct sequencing. Also, to determine whether there is any association between the SNP of the *MMP9* rs17576 gene with chronic generalized periodontitis as measured by PCR restriction fragment length polymorphism (RFLP).

## Materials and methods

Subject selection

A cross-sectional study was carried out over a period of one year and six months in the Department of Periodontics, Government Dental College, Thiruvananthapuram, in collaboration with the Department of Human Molecular Genetics at the Rajiv Gandhi Centre for Biotechnology, Thiruvananthapuram. The study focused on exploring the association between the *MMP9 *gene polymorphism and chronic periodontitis among individuals from a South Indian population. A total of 80 subjects with chronic periodontitis and 90 healthy controls, all of Kerala origin by birth and domicile, were recruited from the outpatient clinic of the Department of Periodontics between March 2013 and September 2014.

Subjects diagnosed with chronic generalized periodontitis, as defined by the 1999 International Workshop for the Classification of Periodontal Diseases and Conditions by the American Academy of Periodontology [15], were included as cases. Individuals with a clinical attachment loss (CAL) greater than 3 mm and involving more than 30% of sites were classified as having chronic generalized periodontitis. Systemically healthy individuals within the same age group, exhibiting a CAL of less than 3 mm, were selected as controls. Exclusion criteria included the presence of systemic or active infectious diseases, pregnancy or lactation, history of smoking, tobacco or pan chewing, alcohol consumption, current medication use, and any oral hard or soft tissue diseases other than dental caries or periodontal disease.

The study received approval from the Institutional Ethical Committee and Review Board of Government Dental College, Thiruvananthapuram (IEC/C/RP-46/2012/DCT, dated December 18, 2012). A structured proforma was used to document participants’ personal information, periodontal status, and the frequency of SNPs at the gene loci under investigation. Following informed consent, blood samples were collected from all participants, and relevant clinical parameters were recorded.

Determination of periodontal status

The clinical parameters assessed were simplified oral hygiene index, plaque index, gingival index, probing pocket depth (PPD), and CAL. PPD and CAL measurements were performed using a William’s graduated probe at six sites per tooth.

DNA isolation and genotyping

**Figure 1:**
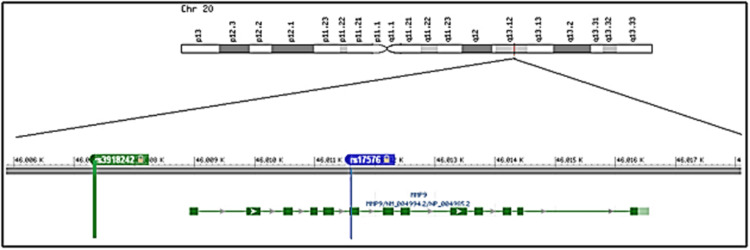
Representation of rs3918242 and rs17576 on chromosome 20

**Figure 2:**
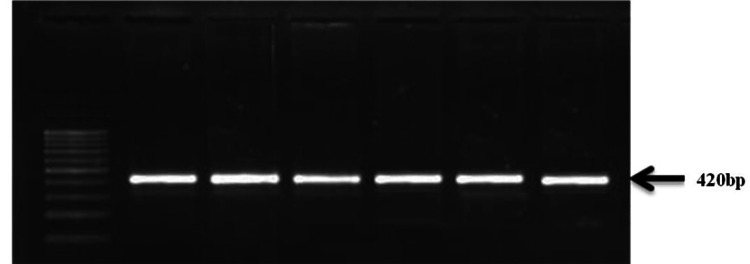
Gel picture showing amplification product obtained with primers for rs3918242

**Figure 3:**
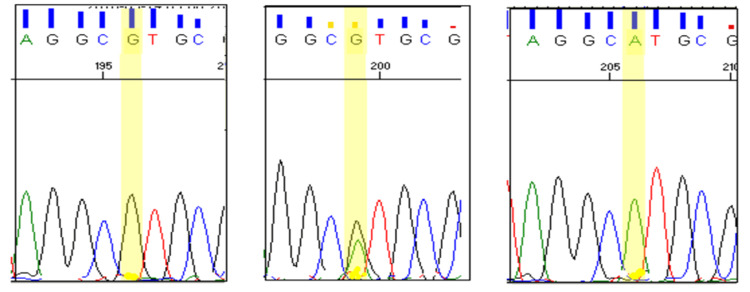
Electropherogram showing homozygous GG, heterozygous GA, and homozygous AA genotypes of the studied SNP rs3918242 (reverse primer was used) SNP: single-nucleotide polymorphism

**Figure 4:**
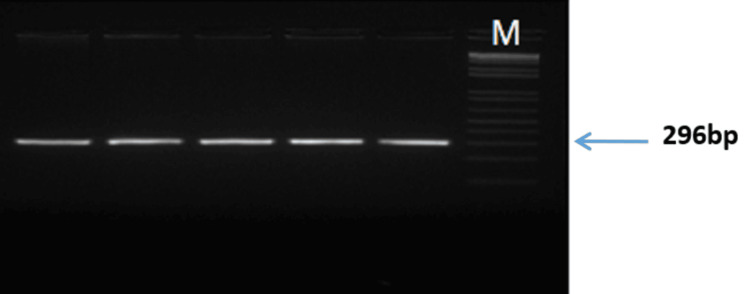
Gel picture showing the amplification product obtained with primers for rs17576

**Figure 5:**
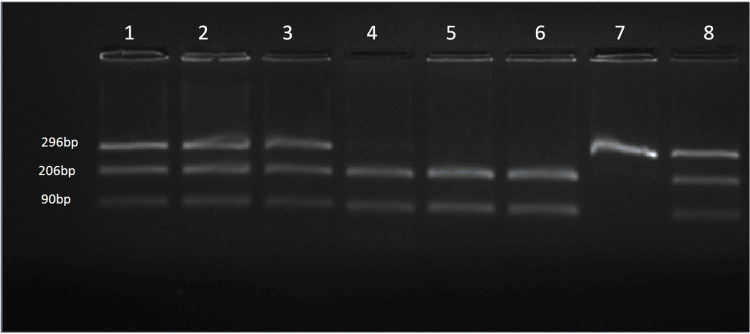
Gel picture showing RFLP of SNP-rs17576 Lanes 4, 5, 6: homozygous AA; Lanes 1, 2, 3, 8: heterozygous AG; Lane 7: homozygous GG RFLP: restriction fragment length polymorphism; SNP: single-nucleotide polymorphism

Peripheral blood samples (5 mL) were collected from study participants via venepuncture after obtaining informed consent. Genomic DNA was extracted from leukocytes using a modified salting-out method [16]. The SNP rs3918242(-1562 C>T) is located in the promoter region, and rs17576 (R+279Q) is located in the exon region of *the MMP9* gene, as shown in Figure 1. PCR was performed using diluted DNA samples and specific primers targeting the regions of interest corresponding to SNPs rs3918242 (-1562 C>T) and rs17576 (R+279Q) of the *MMP9* gene. The amplified product for rs3918242 (Figures 2, 3) was subjected to sequencing PCR using BigDye® Terminator version 3.1 (Thermo Fisher Scientific, Waltham, MA), while the product for rs17576 (Figures 4, 5) was analyzed using RFLP. For the rs17576 SNP, PCR products were digested with the restriction enzyme Sma1. Primer details are provided in Table 1.

**Table 1:** Primers of the MMP9 gene MMP9: matrix metalloproteinase-9

Primer	Sequence 5'-3'	Bases
mmp9rs3918242F	5'-ATgCCTggCACATAgTAggC-3'	20
mmp9rs3918242R	5'-TCgggCAgggTCTATATTCA-3'	20
mmp9rs17576F	5'-ACCATCCATgggTCAAAgAA-3'	20
mmp9rs17576R	5'-gggCTgAACCTggTAgACAg-3'	20

Statistical analysis

Data analysis was performed using the Statistical Package for Social Sciences version 21.0 (IBM Corp., Armonk, NY). Results were presented as frequencies and percentages. The distribution of individual alleles, as well as homozygous and heterozygous genotypes of the *MMP9* gene polymorphisms, was assessed in both case and control groups. Samples that could not be genotyped due to technical issues were excluded from the statistical analysis.

To assess associations and compare variables, the chi-square (χ²) test was employed as a nonparametric test. The odds ratio was calculated for different genotypes and alleles. A p value of <0.05 was considered statistically significant. The genotype frequencies of the *MMP9* rs3918242 (-1562 C>T) and rs17576 (R+279Q), polymorphism in the control population were in Hardy-Weinberg equilibrium.

## Results

The mean age of chronic periodontitis subjects (26 men and 54 women) was 37.2 ± 4.9 years, and that of healthy controls (45 men and 45 women) was 43.9 ± 7.9 years. Table 2 presents the clinical parameters of the subject population.

**Table 2:** Mean of OHI-S, PI, GI, PPD, and CAL in case and control OHI-S: oral hygiene index; PI: plaque index; GI: gingival index; PPD: probing pocket depth; CAL: clinical attachment loss; SD: standard deviation

Parameter	Case (mean ± SD)	Control (mean ± SD)
OHI-S	2.44 ± 0.66	1.41 ± 0.28
PI	1.48 ± 0.36	1.03 ± 0.11
GI	1.81 ± 0.29	0.42 ± 0.24
PPD	3.46 ± 0.52	1.37 ± 0.30
CAL	4.52 ± 0.59	1.63 ± 0.30

In this study, the frequency of the various possible genotypes of the *MMP9* rs3918242 (-1562 C>T) gene polymorphism, i.e., CC, CT, and TT in the cases, was found to be 0.562, 0.411, and 0.027, respectively, and in controls was 0.567, 0.378, and 0.056, respectively. The p value was found to be 0.651 and was not statistically significant. The frequency of the C allele was 0.767 and 0.756 in cases and controls, respectively, while that of the T allele was 0.233 and 0.244, respectively. Again, the p value was not statistically significant at 0.808.

Also, the frequency of the various possible genotypes of the *MMP9* rs17576 gene polymorphism, i.e., AA, AG, and GG, in the cases was found to be 0.164, 0.699, and 0.137, respectively, and in controls was 0.133, 0.556, and 0.311, respectively. The p value was found to be 0.033 and was statistically significant. The frequency of the A allele was 0.514 and 0.411 in cases and controls, respectively, while that of the G allele was 0.486 and 0.589, respectively. Though the p value was not statistically significant at 0.064, it is showing a trend toward significance.

The distribution of genotypes and alleles for *MMP9 *rs3918242 (-1562 C>T) and rs17576 (R+279Q), polymorphisms in both control subjects and individuals with chronic periodontitis, is presented in Tables 3, 4. All genotype distributions conformed to the Hardy-Weinberg equilibrium. While no significant association was found between the -1562 promoter variant of the *MMP9* gene and overall susceptibility to periodontitis, a notable variation in allele distribution was observed within the periodontitis group based on disease severity. The T allele at position -1562 appeared more frequently in patients with severe periodontitis compared to those with mild to moderate disease (p < 0.05). In contrast, no significant differences were observed in the genotype or allele frequencies of the R+279Q variant among the groups.

**Table 3:** Comparison of genotype and allele frequencies of MMP9 rs3918242 (-1562 C>T) between cases and controls A p value of <0.05 was considered statistically significant MMP9: matrix metalloproteinase-9; OR: odds ratio; CI: confidence interval

MMP9		CC	CT	TT	p	C	T	OR (95% CI)	p
rs3918242	Cases	41 (0.562)	30 (0.411)	2 (0.027)	0.651	112 (0.767)	34 (0.233)	0.938 (0.562-1.567)	0.808
	Controls	51 (0.567)	34 (0.378)	5 (0.056)		136 (0.756)	44 (0.244)		

**Table 4:** Comparison of genotype and allele frequencies of MMP9 rs17576 (R+279Q) between cases and controls A p value of <0.05 was considered statistically significant MMP9: matrix metalloproteinase-9; OR: odds ratio; CI: confidence interval

MMP9		AA	AG	GG	p	A	G	OR (95% CI)	p
rs17576	Cases	12 (0.164)	51 (0.699)	10 (0.137)	0.033^*^	75 (0.514)	71 (0.486)	0.661 (0.426-1.026)	0.064
	Controls	12 (0.133)	50 (0.556)	28 (0.311)		74 (0.411)	106 (0.589)		

## Discussion

The assumption that hereditary factors are important in determining the susceptibility and development of periodontitis is supported by a significant number of clinical and scientific data [2]. Research has explored the relationship between chronic periodontitis and various genetic polymorphisms, including those in cytokine genes, metabolism-related genes such as the vitamin D receptor gene, receptor-related genes like the Fcγ receptor gene, MMP genes, and others.

MMP-9 targets denatured collagens (gelatin) and is also active against type IV, V, and XI collagens, proteoglycans, and elastin [10]. The *MMP9* gene is located on chromosome 20q11.2-13.1 and is known to exhibit multiple genetic variants [10]. The promoter genotype of *MMP9* has been associated with several systemic conditions, including complex coronary lesions, heart failure, the initiation and progression of atherosclerosis, and susceptibility to chronic obstructive pulmonary disease.

Several studies have examined MMP polymorphisms in relation to periodontal diseases across various populations, including those by Keles et al. [1], Holla et al. [10], de Souza et al. [17], Chen et al. [18], Gurkan et al. [19,20], Isaza-Guzman et al. [21], Li et al. [22], and Loo et al. [23]. However, the findings have largely been inconclusive, and only a limited number of studies have focused on the Indian population. Therefore, the present cross-sectional study aimed to explore the association between the *MMP9* gene polymorphism and chronic generalized periodontitis in a South Indian population.

In the present study, no statistically significant difference was observed in the distribution of genotypes (CC, CT, and TT) of the *MMP9* rs3918242 (-1562 C>T) polymorphism between chronic periodontitis cases and healthy controls (p = 0.651). The TT genotype, which is thought to enhance *MMP9* transcription, was identified in only five control subjects and two individuals with periodontitis, making it unsuitable for risk estimation. Similarly, no significant difference was found in the allele frequencies of C and T between the two groups (p = 0.808). These findings suggest that the -1562 C>T promoter polymorphism of the *MMP9* gene may not be associated with chronic generalized periodontitis in the study population. However, the lack of association could be attributed to the limited sample size. Therefore, further research with a larger sample is recommended to clarify this potential relationship.

No association was revealed between genotype (p = 0.6935) and allele (p = 0.6693) distribution of the *MMP9* -1562 C>T gene and chronic periodontitis in a study by de Souza et al. [17] in a Brazilian population. The genotype and allele frequencies of *MMP9* -1562 C>T polymorphism in chronic periodontitis and healthy subjects of the Czech population did not vary significantly either (T allele: 0.174 in the healthy group and 0.151 in the chronic periodontitis group, respectively). According to Holla et al. [10], a significant association exists between the severity of chronic periodontitis and the -1562 T allele. When comparing severe and mild forms of chronic periodontitis, the T allele of the *MMP9 *-1562 gene was considerably elevated in the former (0.188 and 0.105, respectively).

In a Turkish population, Gurkan et al. found no notable differences in genotype and allele frequencies of the *MMP9* gene between healthy participants as well as those with chronic periodontitis (p > 0.05) [20]. According to the findings of Isaza-Guzman et al. [21], there was no correlation (p = 0.465) between the various genotypes and chronic periodontitis. The allele frequencies showed a similar pattern (p = 0.869). Keles et al. [1] identified a significantly different genotype distribution among patients having chronic periodontitis as well as healthy controls with regard to the *MMP9 *-1562 C/T polymorphism in a Turkish population, which contrasts with the results of our study. Similarly, a study by Li et al. [22] found that genotype distribution and allele frequency of the *MMP9* promoter gene polymorphism in a Chinese population differed significantly between patients having chronic periodontitis and healthy subjects. According to these findings, the Chinese population's vulnerability to chronic periodontitis was linked to polymorphism in the *MMP9* promoter. The result of our study was in accordance with findings by Sarkar et al. [24] in the Indian population, which revealed no significant association between chronic periodontitis and the *MMP9* -1562 C>T polymorphism.

According to meta-analyses conducted by Pan et al. [25], the *MMP9* -1562 C>T polymorphism has been associated with a reduced risk of chronic periodontitis and appears to influence susceptibility in Caucasian populations. Two meta-analyses [26,27] have evaluated this association. Weng et al. [26] reported that the T allele of the *MMP9* -1562 polymorphism was linked to a lower risk of periodontitis, whereas Mashhadiabbas et al. [27] concluded that there was no significant association in the overall analysis. These findings suggest that the influence of the *MMP9* -1562 C>T polymorphism on periodontitis risk may vary by ethnicity, highlighting the potential role of genetic background in disease susceptibility.

The* MMP9* rs17576 polymorphism is a single-nucleotide variant located within the exon region of the gene. SNP A/G polymorphism of rs17576 in the *MMP9* gene leading to an amino acid substitution in the catalytic domain of the MMP-9 enzyme has been described by Zhang et al. [28], although few functional data are available. Polymorphism at *MMP9* rs17576 has been associated with a higher risk for chronic obstructive pulmonary disease in smokers, lung cancer with metastasis, and myocardial infarction.

In our study, there was a statistically significant difference (p = 0.033) between the frequency of the various possible genotypes of the *MMP9* rs17576 gene polymorphism, i.e., AA, AG, and GG, between the cases and controls. There was no statistically significant difference (p = 0.064) between the frequency of the A and G alleles between cases and controls. The results of the present study suggest that there might be an association between the genotype of *the MMP9* rs17576 gene polymorphism and chronic generalized periodontitis. Though a significant association was not obtained in allele frequency, it shows a trend (p = 0.064) toward association.

Our study's findings are consistent with those of Zorina et al. [29], who used real-time PCR to analyze gene polymorphism and identify a marker panel for the Russian population's propensity for aggressive periodontal disease. In their study, a significant difference was found for gene *MMP9* in the rs17576 position for the A allele (0.552 in aggressive periodontal disease patients and 0.695 in the control group).

In contrast to our study, there were no significant differences in *MMP9* R279Q (rs17576) genotype or allele frequencies between chronic periodontitis and healthy subjects in an investigation by Holla et al. [10] in the Czech population.

To the best of our knowledge, there are only a few studies in the literature, and this is the first study in the Indian population, about the association of the polymorphism of *MMP9* rs17576 with susceptibility to chronic periodontitis. In the present study, the lack of association between the allele frequency of *MMP9* rs17576 and chronic periodontitis might be due to the smaller sample size and the strict guidelines followed for recruiting patients. An analysis with more patients would have greater statistical power and precision. So, further research in this locus with larger samples and a more precise case definition of phenotypes can shed light on this new avenue, which could help us to understand the role of genetic factors in the susceptibility to periodontal diseases.

The powering of studies to reveal an association between multiple SNPs and periodontal disease would probably involve thousands of subjects rather than, at best, the hundreds currently used. Therefore, it is crucial that these findings be replicated in a separate, larger population. Because of the relatively small number of subjects in the study groups, the data should be regarded as a preliminary, hypothesis-generating exercise. To confirm or refute these findings and broaden our observations, functional analysis is suggested, taking into account carriage of a specific combination of alleles within a given locus (haplotype analysis) and among various genes (gene-gene interaction).

The foundation for genetic testing that might be useful in clinical testing may come from the identification of particular genes that contribute to disease susceptibility. Genetic tests may prove useful for identifying patients who are most likely to develop periodontal disease, suffer from recurrent disease, or suffer tooth loss because of periodontal disease. It has been established that, in addition to hereditary variables, environmental risk factors are crucial for the manifestation of disease. For those who are most susceptible to the disease, environment-based preventive and treatment strategies might be developed using the knowledge of the existence of these particular genetic risk factors [30].

In periodontal practice, screening tests are not yet routinely employed. Early detection of patients at increased risk facilitates prevention and early interventional efforts. It may be possible to reduce tooth mortality by routinely monitoring subjects who exhibit a genetic risk factor through a suitable recall and maintenance program. Henceforth, with the understanding of the exact nature of the association of such *MMP* gene polymorphisms with periodontitis, genetic tests can be developed in the future with implications in diagnosis, prevention, and treatment of periodontal disease.

## Conclusions

Significant attempts have been made in recent years to uncover the underlying causes of periodontitis. Although it was believed that pathogenic microflora and other environmental risk factors were part of the pathophysiology of many diseases, there was also compelling evidence that genetic factors were a significant contributor. Through distinct cell stimulation pathways, the bacterial pathogens that cause periodontal disease partially cause damage by triggering a range of proinflammatory cytokines as well as inflammatory mediators from diverse host cells. Among these, the MMP pathway is considered particularly important, as elevated levels of active collagenases and gelatinases have been detected in gingival crevicular fluid, saliva, and tissue biopsies from patients with periodontitis, compared to healthy individuals.

Within the limitations of sample size and selection, the present study suggests no association between the *MMP9* promoter gene polymorphism and susceptibility to chronic generalized periodontitis in the studied population. However, a potential association was observed between the genotype of *MMP9* rs17576 and disease susceptibility, though no significant link was found at the allele frequency level-likely due to the small sample size. Further multicenter studies with larger, age- and gender-matched cohorts are warranted. Such research could help establish the role of *MMP* gene polymorphisms in disease susceptibility and support the development of targeted diagnostic tools and personalized treatment approaches.

## References

[1] Keles GC, Gunes S, Sumer AP, Sumer M, Kara N, Bagci H, Koprulu H (2006). Association of matrix metalloproteinase-9 promoter gene polymorphism with chronic periodontitis. J Periodontol.

[2] Brew K, Dinakarpandian D, Nagase H (2000). Tissue inhibitors of metalloproteinases: evolution, structure and function. Biochim Biophys Acta.

[3] Ryan ME, Golub LM (2000). Modulation of matrix metalloproteinase activities in periodontitis as a treatment strategy. Periodontol 2000.

[4] Uitto VJ, Overall CM, McCulloch C (2003). Proteolytic host cell enzymes in gingival crevice fluid. Periodontol 2000.

[5] Birkedal-Hansen H (1993). Role of matrix metalloproteinases in human periodontal diseases. J Periodontol.

[6] Ingman T, Tervahartiala T, Ding Y (1996). Matrix metalloproteinases and their inhibitors in gingival crevicular fluid and saliva of periodontitis patients. J Clin Periodontol.

[7] Smith PC, Muñoz VC, Collados L, Oyarzún AD (2004). In situ detection of matrix metalloproteinase-9 (MMP-9) in gingival epithelium in human periodontal disease. J Periodontal Res.

[8] Ejeil AL, Igondjo-Tchen S, Ghomrasseni S, Pellat B, Godeau G, Gogly B (2003). Expression of matrix metalloproteinases (MMPs) and tissue inhibitors of metalloproteinases (TIMPs) in healthy and diseased human gingiva. J Periodontol.

[9] Sorsa T, Uitto VJ, Suomalainen K, Vauhkonen M, Lindy S (1988). Comparison of interstitial collagenases from human gingiva, sulcular fluid and polymorphonuclear leukocytes. J Periodontal Res.

[10] Holla LI, Fassmann A, Muzík J, Vanek J, Vasku A (2006). Functional polymorphisms in the matrix metalloproteinase-9 gene in relation to severity of chronic periodontitis. J Periodontol.

[11] Teng YT, Sodek J, McCulloch CA (1992). Gingival crevicular fluid gelatinase and its relationship to periodontal disease in human subjects. J Periodontal Res.

[12] Söder B, Jin LJ, Wickholm S (2002). Granulocyte elastase, matrix metalloproteinase-8 and prostaglandin E2 in gingival crevicular fluid in matched clinical sites in smokers and non-smokers with persistent periodontitis. J Clin Periodontol.

[13] Zhang B, Ye S, Herrmann SM (1999). Functional polymorphism in the regulatory region of gelatinase B gene in relation to severity of coronary atherosclerosis. Circulation.

[14] Mäkelä M, Salo T, Uitto VJ, Larjava H (1994). Matrix metalloproteinases (MMP-2 and MMP-9) of the oral cavity: cellular origin and relationship to periodontal status. J Dent Res.

[15] Armitage GC (1999). Development of a classification system for periodontal diseases and conditions. Ann Periodontol.

[16] Miller SA, Dykes DD, Polesky HF (1988). A simple salting out procedure for extracting DNA from human nucleated cells. Nucleic Acids Res.

[17] de Souza AP, Trevilatto PC, Scarel-Caminaga RM, de Brito RB Jr, Barros SP, Line SR (2005). Analysis of the MMP-9 (C-1562 T) and TIMP-2 (G-418C) gene promoter polymorphisms in patients with chronic periodontitis. J Clin Periodontol.

[18] Chen D, Wang Q, Ma ZW (2007). MMP-2, MMP-9 and TIMP-2 gene polymorphisms in Chinese patients with generalized aggressive periodontitis. J Clin Periodontol.

[19] Gürkan A, Emingil G, Saygan BH, Atilla G, Cinarcik S, Köse T, Berdeli A (2007). Matrix metalloproteinase-2, -9, and -12 gene polymorphisms in generalized aggressive periodontitis. J Periodontol.

[20] Gürkan A, Emingil G, Saygan BH, Atilla G, Cinarcik S, Köse T, Berdeli A (2008). Gene polymorphisms of matrix metalloproteinase-2, -9 and -12 in periodontal health and severe chronic periodontitis. Arch Oral Biol.

[21] Isaza-Guzmán DM, Arias-Osorio C, Martínez-Pabón MC, Tobón-Arroyave SI (2011). Salivary levels of matrix metalloproteinase (MMP)-9 and tissue inhibitor of matrix metalloproteinase (TIMP)-1: a pilot study about the relationship with periodontal status and MMP-9(-1562C/T) gene promoter polymorphism. Arch Oral Biol.

[22] Li G, Yue Y, Tian Y (2012). Association of matrix metalloproteinase (MMP)-1, 3, 9, interleukin (IL)-2, 8 and cyclooxygenase (COX)-2 gene polymorphisms with chronic periodontitis in a Chinese population. Cytokine.

[23] Loo WT, Wang M, Jin LJ, Cheung MN, Li GR (2011). Association of matrix metalloproteinase (MMP-1, MMP-3 and MMP-9) and cyclooxygenase-2 gene polymorphisms and their proteins with chronic periodontitis. Arch Oral Biol.

[24] Sarkar P, Muthuraj TS, Bandyopadhyay P, Ghosh P (2022). Matrix metalloproteinase-9-1562 C/T promoter gene polymorphism in chronic periodontitis: a cross-sectional observational study. J Indian Soc Periodontol.

[25] Pan Y, Li D, Cai Q, Zhang W, Ma J, Wang M, Wang L (2013). MMP-9 -1562C>T contributes to periodontitis susceptibility. J Clin Periodontol.

[26] Weng H, Yan Y, Jin YH, Meng XY, Mo YY, Zeng XT (2016). Matrix metalloproteinase gene polymorphisms and periodontitis susceptibility: a meta-analysis involving 6,162 individuals. Sci Rep.

[27] Mashhadiabbas F, Neamatzadeh H, Foroughi E, Dastgheib SA, Farahnak S, Nasiri R, Ahmadi S (2019). Association of MMP-2-753C>T and MMP-9-1562C>T polymorphisms with chronic/aggressive periodontitis risk: a systematic review and meta-analysis. Iran J Public Health.

[28] Zhang B, Henney A, Eriksson P, Hamsten A, Watkins H, Ye S (1999). Genetic variation at the matrix metalloproteinase-9 locus on chromosome 20q12.2-13.1. Hum Genet.

[29] Zorina OA, Boriskina OA, Rebrikov DV (2013). Correlation of gene polymorphism and risk of aggressive periodontal disease. [Article in Russian]. Stomatologiia (Mosk).

[30] Ottman R (1995). Gene-environment interaction and public health. Am J Hum Genet.

